# THE IMPACT OF THE MELD SCORE ON LIVER TRANSPLANT ALLOCATION AND RESULTS: AN INTEGRATIVE REVIEW

**DOI:** 10.1590/0102-6720201700010018

**Published:** 2017

**Authors:** Ana Claudia Oliveira de MORAES, Priscilla Caroliny de OLIVEIRA, Olival Cirilo Lucena da FONSECA-NETO

**Affiliations:** Instituto Israelita Albert Einstein, São Paulo, SP, Brazil.

**Keywords:** Liver transplantation, Survival analysis, End-stage liver disease

## Abstract

**Introduction:**

Liver transplantation is intended to increase the survival of patients with chronic liver disease in terminal phase, as well as improved quality of life. Since the first transplant until today many changes have occurred in the organ allocation system*.*

**Objective:**

To review the literature on the Model for End-stage Liver Disease (MELD) and analyze its correlation with survival after liver transplantation.

**Method:**

An integrative literature review in Lilacs, SciELO, and Pubmed in October 2015, was realized. Were included eight studies related to the MELD score and its impact on liver transplant.

**Results:**

There was predominance of transplants in male between 45-55 y. The main indications were hepatitis C, hepatocellular carcinoma and alcoholic cirrhosis. The most important factors post-surgery were related to the MELD score, the recipient age, expanded donor criteria and hemotransfusion.

**Conclusion:**

The MELD system reduced the death rate in patients waiting for a liver transplant. However, this score by itself is not a good predictor of survival after liver transplantation.

## RESUMO

**Introdução:**

O transplante de fígado tem como finalidade o aumento da sobrevida dos pacientes com doença hepática crônica em fase terminal, além de melhora na qualidade de vida. Desde o primeiro transplante até os dias atuais, muitas mudanças ocorreram no sistema de alocação de órgãos.

**Objetivo:**

Analisar o conhecimento produzido sobre o Model for End-stage Liver Disease (MELD) e a sua relação com a sobrevida no pós-transplante de fígado.

**Método:**

Realizou-se revisão integrativa nas bases de dados Lilacs, SciELO e Pubmed no mês de outubro de 2015. A amostra contou com oito estudos relacionando o escore MELD e o seu impacto no transplante de fígado.

**Resultados:**

Houve predomínio dos transplantes realizados em homens e faixa etária entre 45-55 anos. Como principais indicações tem-se hepatite C, hepatocarcinoma e cirrose por álcool. Os fatores que tiveram maior impacto no pós-operatório estão associados ao alto valor do MELD, idade do receptor, critérios expandidos do doador e hemotransfusão.

**Conclusão:**

O sistema MELD reduziu a mortalidade na fila de espera, mas isoladamente não é um bom preditor de sobrevivência no pós-transplante de fígado.

## INTRODUCTION

The aim of a liver transplant is to increase the survival of a patient with end-stage liver disease and to improve quality of life^15^. Since the first transplant, in 1963, many changes have significantly improved its success rate^18^. 

The organ and tissue allocation system in Brazil has been through various phases that have led to better organization and greater credibility, reducing waiting lists and hence mortality^1^.

In 1997, the National Organ Transplant and Notification and Distribution Centers System was set up, which enabled the introduction of a Single Technical Register and a single waiting list^1^. At this time, the criterion used for conducting a transplant was time. However, it was noted that mortality on the waiting list was not related only to the waiting time^3^ ^,^ ^10^.

Described for the first time in 2000, the aim of the MELD (Model for End-stage Liver Disease) score was to predict the three-month survival rate in patients who underwent a transjugular intrahepatic portosystemic anastomosis^1^ ^,^ ^3^ ^,^ ^8^. 

A 2001, study validated the MELD score as a measure of the probability of mortality within three months in transplant patients with end-stage chronic liver disease. In 2002, the United States started using this score as a criterion for liver allocation^13^ ^,^ ^26^.

A logarithmic calculation involving serum creatinine, bilirubin and International Normalized Ratio [0.957 x Log e (creatinine mg/dl) + 0.378 x Log e (bilirubin mg/dl) + 1.120 x Log e (INR) + 0.643 x 10, rounded off to the nearest integer] is used to obtain the MELD score for recipients aged over 12 years. It is thus possible to obtain a good predictor of mortality, with a score close to 40 indicating minimal likelihood of survival within three months^1^ ^,^ ^5^ ^,^ ^8^.

The MELD as indicator of the severity of the clinical status of the recipient was introduced as a criterion in Brazil in 2006 by Decree 1160. Since then, a policy has been adopted of using this procedure in more seriously ill patients, with the exception of emergencies and prioritized liver transplants, where allocation is conducted using other criteria, in accordance with current legislation^1^ ^,^ ^4^ ^,^ ^15^ ^,^ ^27^.

This change brought about a reduction of 3,5% in waiting list mortality, an increase of 10.2% in deceased donor transplants and a drop of 12% in patients scheduled for transplant ^3,15^.

Examination of post-transplant survival showed that there may be a relation not defined by a high MELD score alone, but associated with the scarcity of organs, which led to the expansion of criteria for deceased donors beyond comorbidities.

The present study thus aims to examine the information produced on the MELD and its relation to survival and is guided by the following question: What impact does the MELD have on liver allocation and the results of liver transplants?

## METHODS

A bibliographical survey was carried out in October 2015 using three indexed databases: Literatura Latino-Americana em Ciências da Saúde (Lilacs), the Scientific Eletronic Library Online (SciELO) and Pubmed. The articles were searched for using headings controlled by the Virtual Health Library by way of the Health Sciences Headings "liver transplant," "survival analysis" and "end-stage liver disease", and the MESHs (Medical Subject Headings) "liver transplantation," "survival analysis" and "end-stage liver disease".

The selection criteria for articles stipulated that they be available in full, in English, Spanish or Portuguese and published between 2010 and 2015.

The initial search found 76 articles, 49 on Pubmed, 13 on Lilacs and 14 on SciELO. A reading of available titles and abstracts led to the exclusion of 57 articles, 40 from Pubmed, eight from Lilacs and nine from SciELO. Seven of the remaining articles were excluded for being duplicates, leaving 12 articles for analysis. 

These remaining selected articles were evaluated using a methodologically rigorous tool adapted from the Critical Appraisal Skills Programme (CAPS)^21^ containing questions on the clarity of the objective, the adequacy of the methodology, theoretical and methodological procedures, sample selection, the relation between researcher and subject, ethical considerations, rigor and the foundation of data analysis, declaration of results, and the importance of the research. 

Each item was worth one point and the score was the sum of points. Articles with a score of six to ten were classified as being of good methodological quality and low bias (level A)^21^ and were kept in the sample.

**FIGURE 1:**
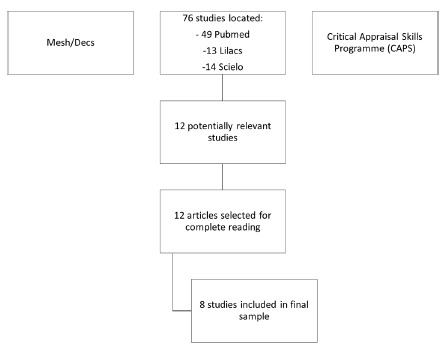
Flowchart of article selection process

Using this tool, the 12 articles were selected and read in full. Eight answered the guiding question and were included in the final sample of the present review: four were from Pubmed, two from Lilacs and two from SciELO (Figure 1).

## RESULTS

**FIGURE 2:**
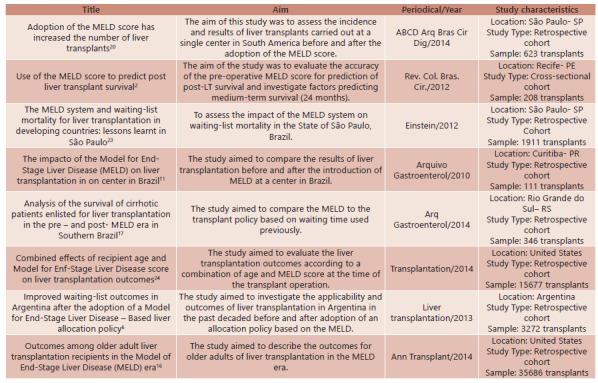
Studies of the impact of the MELD on liver allocation and outcomes of liver transplantation included in the integrative review

Most articles described retrospective cohort-type studies. Figure 2 shows the selected articles and publications.

Most of the liver transplant patients in the articles were male and middle-aged (45-55 years). Most of the studies compared the pre- and post-MELD eras and the inclusion criteria were age ≥18 years, deceased donor transplants, and, in two studies, dual liver-kidney transplant. 

Cases with incomplete medical records and those involving re-transplants, inter-vivo transplants, acute liver failure as an indication, children, multiple organs, and "split-liver" or "domino" operations were generally excluded. Only one study did not include patients with a special MELD score, such as those with hepatocarcinoma^24^ and one other^16^ examined only cases involving patients aged over 50. 

The main indications were viral hepatitis^2,11,17,20,23^ (especially hepatitis C) and alcoholic cirrhotic hepatocellular carcinoma. Patients with hepatocellular carcinoma were selected using the Milan criterion^5^, which classifies cirrhotic patients with a single nodule of up to 5.0 cm in diameter or up to three of up to 3.0 cm in diameter according to the absence of neoplastic thrombosis of the portal system and the absence of extra-hepatic lesions. 

The use of the MELD score as a criterion for liver allocation was found to increase the number of transplantations in cases of hepatocellular carcinoma up to eight-fold^23^.

As expected, a reduction in waiting-list mortality was confirmed by all studies^2^ ^,^ ^6^ ^,^ ^11^ ^,^ ^16^ ^,^ ^17^ ^,^ ^20^ ^,^ ^23^ ^,^ ^24^. However, analysis of postoperative survival revealed that this score is not a good predictor in isolation. This conclusion varied according to the length of time after the transplant surgery but the results were similar for the postoperative period as a whole. 

In cases of dual (liver-kidney) transplants, the short-term survival rate was similar but, in the long term, a single (liver) transplant had a better outcome^20^. Hepatocellular carcinoma did not correlate with a worse survival rate and cases that were not in an advanced stage were found to be associated with lower postoperative morbidity and mortality^11^ ^,^ ^17^. One study^16^ found hepatocellular carcinoma to be a risk factor for higher mortality within five years.

Factors that had a postoperative impact and worse survival rates included: delay between supply and demand, which may cause the MELD score to increase while waiting for the transplant, producing a more severe condition at the time of the surgery and hence a greater likelihood of complications; age over 60 years; pre-transplant dialysis; the use of broader criteria to select donors; and transfusion of blood products. 

## DISCUSSION

In terms of age and the predominance of male transplant patients, the findings of the various studies were similar^3^ ^,^ ^9^ ^,^ ^14^ ^,^ ^15^. Waiting-list mortality and exclusion for reason of worsening of the underlying condition are more common among women, possibly because of lower creatinine levels. This shows that women are at a disadvantage under a MELD-based allocation system^15^. 

Hepatitis C is the main indication for liver transplantation and HCV recidivism, which is considered the most common cause of graft loss, occurs in 90% of patients in the first year after surgery^22^. 

The Ministry of Health defines cases of hepatitis C confirmed by positive anti-HCV and HCV-RNA as detectable. The detection rate is high and more common in patients aged between 30 and 59 years and has been reported by Sharpton et al.^24^ as the main indication for transplantation in young patients^14^. 

The MELD era has seen an increase in transplantation for hepatocellular carcinoma and fewer patients excluded from the waiting list^15^ ^,^ ^17^ ^,^ ^23^. In many of these cases, liver functioning is fairly well preserved and this leads to a lower MELD score, a longer waiting time and worsening of the patient's condition. Additional points are thus added to the score to compensate for the higher risk of being without criteria for liver transplantation for reason of a tumor or metastasis, benefiting these patients without disadvantaging other cases. It is worth noting that the waiting time for transplantation has shortened considerably in MELD era, leading to higher survival rates^1^ ^,^ ^5^ ^,^ ^15^ ^,^ ^23^ ^,^ ^28^.

The three-month increase in waiting-list survival was confirmed by the present review but, after surgery, the best outcomes are associated with a low MELD score. In Machado^15^, various studies attempted to relate this score to the post-transplant period and establish a cut-off point to guide the decision not to perform transplantation, despite the worst outcomes occurring with the higher MELD scores. But this was not possible^7^.

Cases of dual (liver-kidney) transplants increased during the MELD era and there was not much difference in short-term survival rate between this procedure and liver transplant alone. Veras et al.^25^ came up with a similar finding but also noted a lower survival rate in the 3^rd^ and 5^th^ years after a dual transplant. 

Furthermore, cases of pre-operative kidney dysfunction are more likely to result in the need for dialysis after transplantation, primary liver graft dysfunction and death. This suggests that a dual transplant may benefit these recipients^19^ ^,^ ^25^.

Although the MELD score is not directly related to transplantation outcomes, some features are better correlated with the postoperative scenario. Advanced age may lead to early loss of the graft, especially when combined with a high MELD score, with almost half of these transplant patients with a MELD ≥28 dying in the first year^24^. This may also be due to comorbidities associated with age.

Blood transfusion is also linked to reduced survival after transplantation^2^ ^,^ ^11^, with an association between multiple transfusions and complications seen as a predictor of survival^3^. 

This may be related to immunomodulation related the transfusion of blood products and also by the storage process resulting in an increase in hospital infections, acute lung damage and the development of autoimmune diseases in the long term. The need for blood transfusion may also be related to the severity of the condition of the patient at the time of the transplant surgery, in which cases a lower survival rate was already expected^2^ ^,^ ^19^.

Another important issue is the scarcity of organs, which leads to the broadening of criteria used to select donors around the world with good results. However, some factors relating to the donor, such as age, race, cardiac arrest, stroke, cold ischemia time and split liver are related to early graft failure, especially in the case of the age of the donor^2^ ^,^ ^12^ ^,^ ^16^. 

The factors that have the greatest impact on liver transplantation survival are not directly linked to the severity score used in Brazil since 2006. Furthermore, the intrinsic limitations of the MELD and those associated with the clinical condition of the patient have led to the incorporation of new variables to improve its predictive capacity^15^. 

## CONCLUSION

The change to the MELD system of allocation has increased the number of transplants and led to lower waiting-list mortality. However, it has not had a significant impact on post-transplant outcomes or survival. A combination of the MELD score and other preoperative factors may provide a better indication of the likelihood of complications and allow for better assessment of the risks of the operation. As yet there is no score that better predicts the outcomes of surgery but it is clear that MELD alone is not a good predictor of survival subsequent to liver transplant surgery. 
